# Identification of a Sialosides binding pocket in early-lineage SARS-CoV-2 via an optimized STD NMR method: a novel explanation for coronavirus virulence and zoonosis

**DOI:** 10.1186/s43556-022-00093-0

**Published:** 2022-10-21

**Authors:** Geng Chen, Wei Gao, Yang Du

**Affiliations:** 1 Kobilka Institute of Innovative Drug Discovery, School of Medicine, The Chinese University of Hong Kong, Shenzhen, 518172 Guangdong China; 2 The Chinese University of Hong Kong, Shenzhen Futian Biomedical Innovation R&D Center, Shenzhen, China

Recently, Benjamin G. Davis group in Rosalind Franklin Institute and Oxford university revealed a novel interaction between sugars on the human cell surface and SARS-CoV-2 (“severe acute respiratory syndrome coronavirus 2) spike protein in *Science* by an optimized saturation transfer difference (STD) NMR method that named as universal STA [1].

It has been three years since Covid-19 became a global pandemic and it seems that SARS-CoV-2 will co-exist with human beings for a long time in future. Although it has been clearly demonstrated that SARS-CoV-2 enters human body via the classic binding between the spike protein receptor-binding domain (RBD) and human angiotensin converting enzyme-2 (ACE2) and the recognition mechanism was extensively studied [2], the interaction between SARS-CoV-2 spike protein and glycans on the human cell surface remains unclear. In this paper, the authors speculate that the N-term domain (NTD) of the spike protein in B-origin lineage SARS-CoV-2, which located adjacent to the RBD, can bind to Sialosides and thus influence the virulence and zoonosis of the virus. Besides, this binding is independent of the RBD-ACE2 interaction but may work cooperatively with it in the infection process (Fig. 1a).

**Fig. 1:**
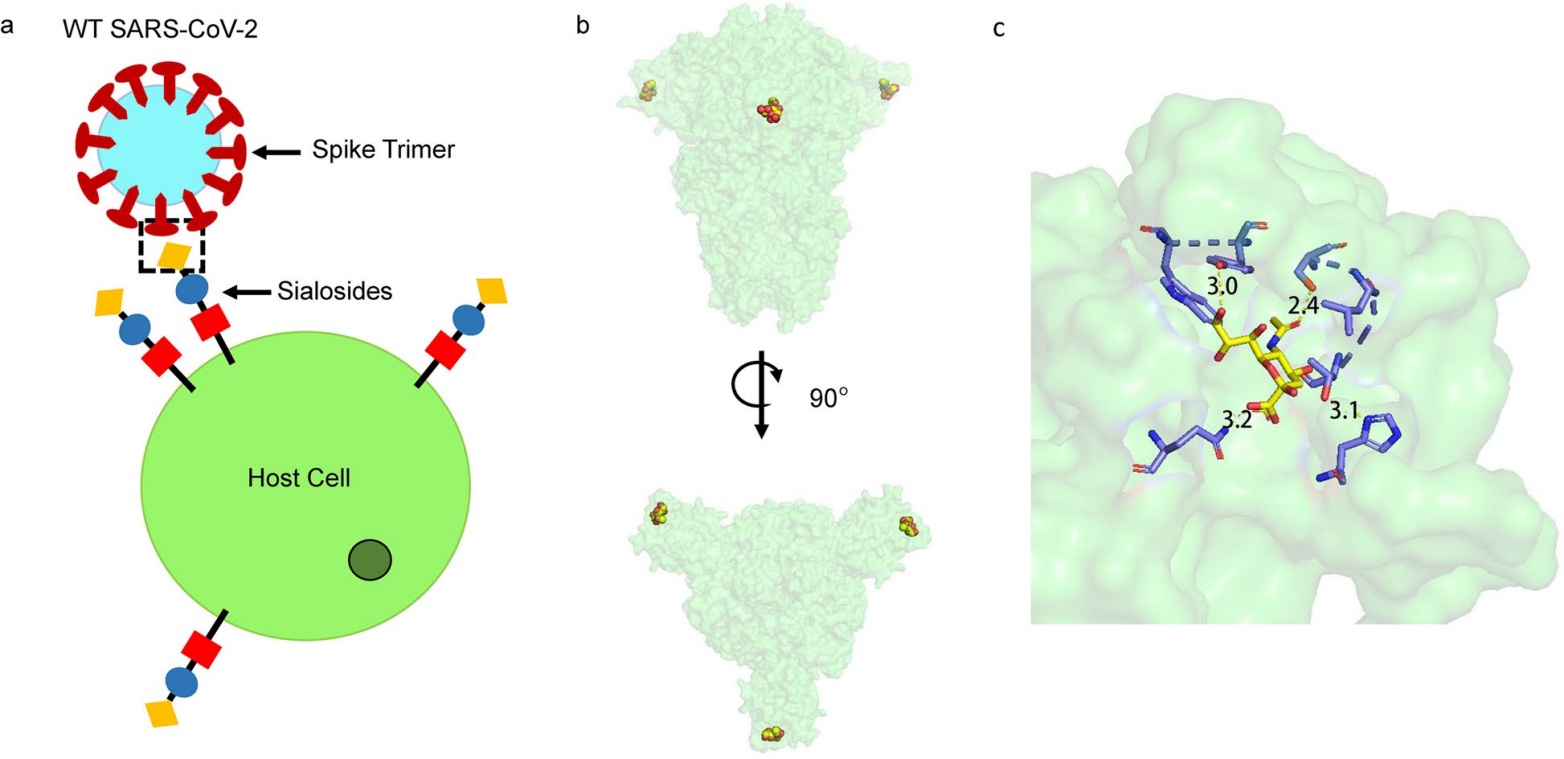
The molecular mechanism of SARS-CoV-2 spike protein NTD domain interacts with Sialosides on human cell surface. **a** Graphical representation showing host–pathogen interactions. **b** Cryo-EM structure of spike protein that binds Sialosides to via its NTD, which located at the outer surface of spike protein. Sialosides (yellow) are shown in dots and the spike trimer (green) are shown in surface. **c** A close look to the binding site for Sialosides. The pocket is formed by H69, Y145, W152, S247 and Q183 in the spike NTD. Hydrogen bonds are show in yellow dash lines

Sialosides are a class of 9 carbon carboxylated monosaccharide derivatives, existing as short chain residues attached to the end of glycolipid and glycoprotein through α-2,3、α-2,6 or α-2,8 linkages. Sialosides play critical roles in viral and bacterial infections, for example, the interaction between hemagglutinin (HA) protein trimer on the surface of influenza viruses and Sialosides on the surface of host cell promotes viral attachment and consequent viral entry [3]. Furthermore, this binding is also observed in non-SARS coronaviruses including Middle East respiratory syndrome coronavirus (MERS-CoV), a close relative of SARS-CoV-2 [4]. The spike protein of SARS-CoV-2 has a structurally similar NTD with a putative Sialosides binding pocket [5], however, there is no clear clue that how its NTD works during infection process and the function of it remains confused. Charles et al. confirmed the interaction between spike NTD and Sialosides by using a new NMR method. They modified existing STD NMR through theoretical analysis and computational approach and overcome the rigorous constraint on binding rates for protein–ligand interaction, hence expanding its application. This altered method was named as uSTA due to the improved compatibility. Combined uSTA and Cryo-EM results demonstrated that spike NTD harbours a binding site for Sialosides that formed by H69, Y145, W152, S247 and Q183, which located on the outer surface of spike protein (Fig. 1b, c). Sialosides binds to SARS-CoV-2 spike in an ‘end-on’ manner, and interestingly, despite the high sequence similarity with MERS-CoV, the structural superposition revealed a 12 Å distance between two sialic acid binding pockets. Besides, the secondary structure elements used for binding are different: in MERS spike structure (RCSB 6NZK) sialic acid located at the edge of the central β-sheet, whereas in this newly identified SARS-CoV-2 spike structure it is bound at the centre of the β-sheet. However, this feature disappeared in the subsequent alpha, beta, delta and Omicron variants. Theamino acids around this pocket were mutated in the SARS-Cov-2 variants and therefore lose the ability to bind Sialosides on the lung cell surface, which may facilitate the release of virus from cells of the respiratory stystem and increase the infectivity as we observed in the epidemiologic studies.

Considering that Sialosides attach on cell surfaces as glycol-conjugates of glycolipid and glycoprotein, the authors assumed that glycosylation function in human body also impacts on SARS-CoV-2 infection. By analysing whole exome sequencing data derived from 533 patients infected with SARS-CoV-2, the authors identified two genes (LGALS3BP and B3GNT8) related to glycan among the top five which affect disease severity. Genetic variants of these two genes are related to a decrease in severe disease outcome. These two genes are associated with N-linked-polyLacNAc-chains, and therefore the authors indicate that SARS-CoV-2 utilizes N-linked-polyLacNAc-chains as an anchor point to attach on host cells. Taken together, genetic analysis of patients from Italy in 2020 revealed that patients with mutations in two specific genes related to the type of cell surface Sialosides exhibit a lower ratio of severe disease. Combined with the previous experiment data, it demonstrated that it is hard for the original virus to enter the deep inside of the lung in patient with altered Sialosides type, resulting in a less severe disease. These observations are consistent with the fact that the virulence is alleviated in new variants including alpha, beta, delta and Omicron.

Altogether, Charles et al. demonstrated the presence of a Sialosides-binding site in NTD of SARS-CoV-2 spike protein and delineate the mechanism underlying the interaction in early B-origin lineage virus. Nevertheless, the Sialosides-binding site is abolished during the spread of SARS-CoV-2. These observations implicate that the ability to bind Sialosides is required for viruses during the spread from intermediate host to human beings; however, this interaction is abrogated to facilitate the release from cell surface and enhance spread ability during pandemic. These observations shed light on the zoonosis, virulence and pandemic mechanism of SARS-CoV-2, and will definitely provide help for the long-term virus evolution study. The new uSTA NMR method developed by Charles et al. could also be utilized to investigate various host–pathogen interactions including influenza viruses, MERS-CoV and other infective microbes on a molecular level.

## References

[1] Buchanan CJ, Gaunt B, Harrison PJ, Yang Y, Liu J, Khan A (2022). Pathogen-sugar interactions revealed by universal saturation transfer analysis. Science.

[2] Shang J, Ye G, Shi K, Wan Y, Luo C, Aihara H (2020). Structural basis of receptor recognition by SARS-CoV-2. Nature.

[3] Li Z, Lang Y, Liu L, Bunyatov MI, Sarmiento AI, de Groot RJ (2021). Synthetic O-acetylated sialosides facilitate functional receptor identification for human respiratory viruses. Nat Chem.

[4] Park YJ, Walls AC, Wang Z, Sauer MM, Li W, Tortorici MA (2019). Structures of MERS-CoV spike glycoprotein in complex with sialoside attachment receptors. Nat Struct Mol Biol.

[5] Awasthi M, Gulati S, Sarkar DP, Tiwari S, Kateriya S, Ranjan P (2020). The Sialoside-Binding Pocket of SARS-CoV-2 Spike Glycoprotein Structurally Resembles MERS-CoV. Viruses.

